# Tuberculosis suspicion and knowledge among private and public general practitioners: Questionnaire Based Study in Oman

**DOI:** 10.1186/1471-2458-8-177

**Published:** 2008-05-26

**Authors:** Abdullah A Al-Maniri, Omar A Al-Rawas, Fatmah Al-Ajmi, Ayesha De Costa, Bo Eriksson, Vinod K Diwan

**Affiliations:** 1 Department of Public Health Sciences, Division of International Health (IHCAR), Karolinska Institutet, Sweden; 2 Department of Family Medicine and Public Health, College of Medicine and Health Sciences, Sultan Qaboos University, Sultanate of Oman; 3 Department of Medicine, College of Medicine and Health Sciences, Sultan Qaboos University, Sultanate of Oman; 4 Directorate General of Health Services of Muscat, Ministry of Health, Sultanate of Oman; 5 Nordic School of Public Health, Gothenburg, Sweden

## Abstract

**Background:**

Early detection of smear positive TB cases by smear microscopy requires high level of suspicion of TB among primary care physicians. The objective of this study is to measure TB suspicion and knowledge among private and public sector general practitioners using clinical vignette-based survey and structured questionnaire.

**Methods:**

Two questionnaires were distributed to both private and public GPs in Muscat Governorate. One questionnaire assessed demographic information of the respondent and had 10 short clinical vignettes of TB and non-TB cases. The second questionnaire had questions on knowledge of TB, its diagnosis, treatment, follow up and contact screening based on Ministry of Health policy. TB suspicion score and TB Knowledge score were computed and analyzed.

**Results:**

A total of 257 GPs participated in the study of which 154 were private GPs. There was a significant difference between private and public GPs in terms of age, sex, duration of practice and nationality. Among all GPs, 37.7% considered TB as one of the three most likely diagnoses in all 5 TB clinical vignettes. Private GPs had statistically significantly lower TB suspicion and TB knowledge scores than public GPs.

**Conclusion:**

In Oman, GPs appear to have low suspicion and poor knowledge of TB, particularly private GPs. To strengthen TB control program, there is a need to train GPs on TB identification and adopt a Private Public Mix (PPM) strategy for TB control.

## Background

Detection of smear positive TB cases by direct microscopy is a key element of the DOTS (Direct Observed Treatment – Short course) strategy [1]. The success of this strategy depends on the ability of the health care system to identify and follow up TB suspects [2]. Health system's inability to diagnose and treat TB has been shown by many studies, both in low and high incidence countries [3-7].

Although Oman has been able to significantly reduce the burden of all forms) TB over the last 25 years, from 85 to 12 cases per 100,000 population. However, a slower decline was observed in the latter 10 years [8]. Along with other Gulf Cooperation Council (GCC) countries, Oman fixed an 'elimination target' for smear positive cases defined as 3/100,000 population by 2005 and 1/100,000 population by 2010. However, the smear positive notification rate in 2005 was 6/100,000 population, which necessitates additional efforts towards TB control in the country including systematic evaluation of the various components and factors affecting TB control [8].

In Oman, modern public health care services are free and delivered at three different levels, primary, secondary and tertiary. Secondary and tertiary hospitals have subspecialties and admission facilities. They are mainly located in the major cities. Primary health care is the main mode of public health care delivery, delivered through 140 health centers all over Oman, manned by general practitioners (GPs). In addition, private health care is available in Oman and has increased over the last 10 years. Private health care is delivered mainly through private clinics/hospitals and private pharmacies. In private clinics and/hospitals, GPs are the main providers for both Omani nationals and non-nationals (guest workers) [9].

Both public and private GPs are at the front line with regard to the diagnosis of TB. Therefore, skillful and adequately trained GPs would facilitate identifying TB cases. However, while both public and private GPs can make a diagnosis of TB, in Oman only public GPs are authorized to follow up and manage TB patients, under the law [10]. Being a rapidly expanding sector, it is important to evaluate the ability of private GPs to suspect TB. The aim of this paper was to study level of suspicion and knowledge of TB among private and public GPs using written vignettes and structured questionnaires.

## Methods

### Study setting

The survey was carried out in Muscat Governorate which hosts the capital of Oman, Muscat city. Muscat governorate has 6 willayates (towns) with approximately 700,000 inhabitants. Each willayate is serviced by 4 to 6 public primary health care centers (depending on population). Most health centers have 8 to 10 GPs. In addition, Muscat has the largest private sector in Oman with more than two thirds of all private health manpower in the country practicing in Muscat.

### Study Participants

All GPs working in both private and public sector in Muscat were targeted in the study. (n = 326; 54.3% private and 45.7% public). Public and private physicians worked in out patient clinics, staffed by an average of 8–10 GPs in the public sector and 2–3 in the private sector. Public GPs were both nationals and non-nationals with a basic medical degree but no specialty training. There were a few family physicians working in health centers and these were excluded from the survey. Private GPs were mostly non-nationals with a basic medical degree.

### Study Materials

The survey instrument comprised two questionnaires (A & B). Questionnaire (A)) See Additional file 1) had two parts. The first part solicited background demographic information of respondents (Table 1). The second part had 10 short written case vignettes (Box 1). Each vignette, described a patient who had key symptoms and signs of a respiratory illness. Some vignettes also included results of diagnostic procedures. Five of the vignettes were TB cases with different presentations. The other five vignettes were typical cases of Chronic Obstructive Pulmonary Disease (COPD), pneumonia, sarcoidosis, bronchiectasis and pulmonary fibrosis. In each of the 10 vignettes, GPs were asked to provide a maximum of 3 possible diagnoses in order of likelihood (i.e. most likely diagnosis first).

**Table 1:** General characteristics of the GPs participating in the study

Characteristic	All	Public	Private
	N= 257	N= 103	n= 154
Age in years (mean)	41.3	35.2	45.2
Professional Experience in years (mean)	15.2	9.3	18.9
Male (%)	46.1	35.1	53.4
Omani (%)	20.3	48.5	2.0
Found/followed up TB case last year (%)	31.9	64.1	10.4
Attended TB training course during the last 5 years (%)	14.2	19.2	11.0

The clinical vignettes were prepared and the diagnoses finalized by a team of chest and infectious disease specialists and medical residents at Sultan Qaboos University Hospital, Muscat.

Questionnaire (B) (See Additional file 2) focused on TB and also had two parts. The first was on experience with TB, i.e. diagnosing and following up TB cases, TB training courses attended, and the second had a case vignette of a classical case of pulmonary TB followed by 20 multiple choice questions (MCQs) pertinent to the diagnosis, treatment, contact screening and follow up of such a TB case. The questions were based on the national TB manual published by Ministry of Health [10]. There was only one correct answer for each question. The questionnaire was also reviewed and finalized by the same group of specialist physicians.

### Data Collection

#### Public sector GPs

Each public health centre has a nurse in-charge. She, along with other nurses moderates patients' movements to consultation rooms of the GPs. We trained all nurses in-charge of all 17 health centers in Muscat governorate. Towards the end of the morning hours, the nurse in charge presented questionnaire (A) first to the available GPs and requested them to seal the questionnaire once they answered it. Then, questionnaire (B) was given to the same GPs and similarly sealed. GPs working in the evening hours were given the questionnaires in a similar manner.

#### Private sector GPs

For the private sector, we trained health inspectors who were familiar with all the private clinics in Muscat (they routinely visit them every week). Health inspectors were asked to give the questionnaire to all available private GPs. The questionnaires were distributed in a similar way to the public GPs i.e. questionnaire (A) first and then (B). The health inspectors were asked to wait in the clinic until the GPs finished responding to both questionnaires.

### Statistical Analysis

A TB suspicion score in the five TB vignettes was computed for each GP. We awarded 3, 2, 1 and 0 points for those who gave TB as a first, second, third and not-considered diagnosis, respectively. We also computed a TB general knowledge score from 20 MCQs (one point for every correct answer and none for an incorrect one). The reliability of the questionnaires was tested using chronbach alpha statistic and it was estimated to be 0.51 for the suspicion questionnaire and 0.64 for the knowledge questionnaire. Standard statistical measures were used to summarize the characteristics of GPs. Statistical test were used to assess the influence of random variation in the comparisons of groups. Comparison between public and private GPs was carried out using Chi square test for categorical variables. Independent sample t-test was used to compare the means of age and experience in years. Mann-Whitney test was applied for comparison of the distributions of the scores. A p-value of 0.05 and less was considered as indicating a statistically significant difference. We used multiple linear regression analysis to study a model with the two kinds of scores as dependent variables and potentially related independent variables. Robust estimation of standard error was used due to possible non-normality and heteroscedastisity of the observed TB suspicion and knowledge scores [11].

### Ethical considerations

Participants were given choice not to participate in the study. Anonymity was assured by not collecting individual details related to name, health center or clinic. The study was approved by the medical research and ethics committee (MREC), Sultan Qaboos University, College of Medicine and Health Sciences, Muscat, Oman

## Results

During the study period, we met 170 (96%) of the private GPs and 117 (78.5%) of the public GPs. Seven of the private GPs were on leave and 32 public GPs were also on leave or away for training courses. Out of the 170 private GPs, 13 (7.6%) refused to participate and 2 returned unanswered questionnaire (1.2%). Among the 117 public GPs, 9 (7.8%) refused to participate and 5 (4.3%) did not return the questionnaire.

Table 1 shows general characteristics of GPs in the study. The average age of the GPs was 41.3 years with an average experience of 15.2 years. Private GPs were 10 years older than the public GPs (45.2 versus 35.2; p < 0.001) and had longer experience (18.9% versus 9.3%; p < 0.001). There were more non-nationals among private than public GPs (98.0% versus 51.5%; p < 0.001). A private GP was less likely to have diagnosed or followed up a TB case in the last year compared to a public GP (10.4% versus 64.1%; p < 0.001). Less than 15% of GPs had attended TB training courses during the last 5 years (19.2% public versus 11.0% private; p = 0.07).

Table 2 shows number and percentage of GPs who considered TB as one of the three possible diagnoses in any of the clinical vignettes. Except for TB-HIV vignette and TB vignette with heamoptysis, the percentage of private GPs who considered TB as one of three likely diagnoses in TB vignettes were significantly lower than public GPs. In non-TB vignettes, there was no significant difference in considering TB except for pneumonia vignette where 21.2 % of public GPs considered TB as compared to 5.5% of private GPs (p < 0.001). Among all GPs, 37.7% considered TB as one of the three most likely diagnoses in all 5 TB clinical vignettes with private having significantly lower percentage compared to public (27.3% versus 53.4%, p value 0.001)

**Table 2:** Numbers and percentages of GPs who considered TB as one of the three possible diagnoses in any of the 5 clinical vignettes describing TB-cases

Clinical Vignette			
	Private	Public	P-Value
	N (%)	N (%)	
TB case 1 (5 weeks cough, loss of weight)	93 (60.4%)	81(78.6%)	0.002
TB case 2 (HIV with prolonged cough)	119 (77.3%)	83 (80.6%)	0.526
TB case 3 (loss of weight, night sweats)	112(72.2%)	91 (88.3%)	0.003
TB case 4 (cough with heamoptysis)	109 (70.8%)	78 (75.7%)	0.382
TB case 5 (Cough, crackles at the apex)	119 (77.3%)	96 (93.2%)	0.001
Sarcoidosis	70 (49%)	44 (44.4%)	0.490
Fibrosis	48 (32.9%)	24 (24.2%)	0.145
Pneumonia	8 (5.5%)	21 (21.2%)	0.001
bronchiectasis	16 (11.1%)	13 (13.1%)	0.633
COPD	13 (9.0%)	12 (12.1%)	0.420

Furthermore, for cases of sarcoidosis, fibrosis, bronchiectasis and COPD, more than 50% of all GPs were not able to mention the correct diagnosis with no significant difference between the public and private GPs. Pneumonia was correctly diagnosed by more than 80% of both public and private GPs (no statistically significant difference was observed).

Table 3 shows the median and inter-quartile range of TB suspicion and TB knowledge scores. The median scores of all GPs were 11 and 8 for suspicion and knowledge, respectively. For both scores private GPs scored significantly lower than public GPs (p < 0.001). Significant difference in knowledge related to diagnosis (p = 0.037), treatment (p < 0.001) and contact screening (p = 0.041) was observed.

**Table 3:** Median and Interquartile range of TB suspicion and TB knowledge Scores.

Score	Public	Private	P-value
	Median (Interquartile range)	Median (Interquartile range)	
TB Suspicion Score (maximum 15)	12.0(9.0–14.0)	10.0(7.0–12.0)	0.001
TB Knowledge Score (maximum 20)	9.0(7.0–11.75)	7.0(5.0–9.0)	0.001
• Diagnosis (maximum 5)	2.0(2.0–3.0)	2.0(1.0–3.0)	0.037
• Treatment (maximum 8)	5.0(3.0–7.0)	3.0(2.0–5.0)	0.001
• Follow up (maximum 4)	1.0(0.0–1.0)	1.0(1.0–1.0)	0.199
• Contacts screening (maximum 3)	1.0(0.0–1.0)	1.0 (0.0–1.0)	0.041

Tables 4 and 5 present the results of simple and multiple robust linear regressions, respectively, with TB suspicion and TB knowledge scores as dependent variables and general characteristics of GPs as independent. "sector of work" (public/private) and "finding or following a TB case last year" were related to TB suspicion score using simple robust regression analysis. However, in the multiple analysis, sector of work was the only variable associated with TB suspicion score with private GPs scoring 1.6 points less than public GPs (95%CI; -2.9, -0.31). In simple analysis, sector of work, nationality, attending TB training course and "finding or following a TB case last year" were independently related to TB knowledge score. In the multiple analysis, private GPs scored 1.5 points less than public GPs (95%CI; -2.74, -0.28). GPs having attended TB training course in the last 5 years scored 2 points more than those who did not 95%CI; (0.71, 3.24).

**Table 4:** Estimated regression coefficients, with 95% confidence intervals in brackets, in simple robust linear regression analyses with TB suspicion score and mean TB knowledge score as dependent variables (95% CI).

Factor4		TB Suspicion Score	TB Knowledge Score
Independent variable		Dependent variable	Dependent variable
Sector	Public	Ref.	Ref.
	Private	-1.71(-2.60, -0.82)*	-2.05(-2.87, -1.23)*

Gender	Female	Ref.	Ref.
	Male	0.30(-0.62, 1.22)	-0.08(-0.94, 0.77)

Nationality	Not-Omani	Ref.	Ref.
	Omani	0.76 (-0.39,1.91)	1.56 (0.47, 2.66)*

Duration of Practice	≤ 5 years	Ref.	Ref.
	> 5 years	-0.32(-1.60, 0.98)	-0.84 (-1.94, 0.26)

Attended TB training course during last 5 years	No	Ref.	Ref.
	Yes	0.67(-0.64, 1.98)	1.69(0.46, 2.91)*

Found/followed up a TB case last year	No	Ref.	Ref.
	Yes	1.36(0.39, 2.32)*	1.74(0.85, 2.62)*

TB Knowledge score		0.17(0.03–0.32)*	Not Applicable

For binary independent variables the coefficients are estimates of the difference between the means at the two levels.

* Statistically Significant.(p-value is less than 0.05)

**Table 5:** Estimated partial regression coefficients, with 95% confidence intervals in brackets, in multiple robust linear regression analyses with TB suspicion score and mean TB knowledge score as dependent variables (95% CI).

Factor		TB Suspicion Score	TB Knowledge Score
Independent variable		Dependent variable	Dependent variable
Sector	Public	Ref.	Ref.
	Private	-1.61(-2.92, -0.31)*	-1.51(-2.74, -0.28)*

Gender	Female	Ref.	Ref.
	Male	0.63(-0.28, 1.55)	0.04(-0.82, 0.90)

Nationality	Not-Omani	Ref.	Ref.
	Omani	-0.10 (-1.86,1.65)	0.97(-0.70, 2.63)

Duration of Practice	= 5 years	Ref.	Ref.
	> 5 years	0.82(-0.99, 2.65)	1.0(-0.48, 2.46)

Attended TB training course during last 5 years	No	Ref.	Ref.
	Yes	-0.33(0.86, 1.65)	1.97(0.71, 3.24)*

Found/followed up a TB case last year	No	Ref.	Ref.
	Yes	0.66(-0.53, 1.86)	0.86(-0.25, 1.96)

TB Knowledge score		0.10(-0.05–0.24)	Not Applicable

For binary variables the coefficients are estimates of the difference between the means at the two levels

* Statistically Significant.(p-value is less than 0.05)

## Discussion

Vignettes or written case simulations have been used widely to assess clinical knowledge and competence, though their use is controversial when assessing practice is concerned [12]. However they were suitable in our study, as our intention was to assess knowledge and competence, and not clinical performance or action. These former two aspects form the base of an elegant conceptual model of clinical competence, Miller's pyramid [13] and lend themselves to evaluation using vignettes. Vignettes have the advantage of being easily administered, less costly and usable across different types of clinical practices. Vignettes are uniquely suited for comparative analyses among different providers/groups because they control for case mix. A study that assessed vignettes in comparison to the gold standard of standardized patients in an out patient setting similar to ours, showed that measuring quality with vignettes consistently produced scores that were close to the standardized patient method [12]. It concluded that vignettes appeared to be most useful when the focus of research was on measuring competence among a group of providers.

Our results indicate that both private and public GPs in Oman are unlikely to suspect TB when TB is one of three likely diagnoses. The reasons of this low suspicion and poor knowledge are not clear. However, a possible explanation of the above results could be that as Oman is already a low incidence country (with less than 6/100,000 smear positive cases reported annually), there is little encounter with TB in clinical experience [7] (which would make GPs consider other, more common, respiratory conditions). Pneumonia and chronic bronchitis were the most common alternative diagnosis to TB (data not shown). There is a lack of TB training courses, less than 15% of all GPs attended TB courses during the last 5 years. A recent randomized controlled trial showed that educational interventions aimed to promote TB screening are effective in increasing TB detection [12].

Further, we have found that private GPs have significantly lower levels of suspicion and lower knowledge of TB compared to public GPs. This is consistent with findings in other studies in high incidence setting, e.g Pakistan and India, and also in low incidence setting, e.g US [14-17]. The fact that public GPs were more involved in finding and following TB cases than private is a possible explanation of the observed difference. Cases identified in the private sector are to be notified and referred immediately to the public health system. A possible implication of this policy is that private GPs lose interest in diagnosing and managing TB. Another possible explanation for the above difference is that public GPs reported attending TB training courses more frequently than private GPs which had an impact on their overall TB knowledge. In addition, public GPs as group of physicians (8 to 10 GPs in each health centre) share together knowledge and experiences to a greater extent than private GPs, where a maximum of 2 at each private clinic practice together.

General practitioners knowledge may have an impact on their patients' health. An important implication of our results is that there can be delays in diagnosis of TB cases, especially among those attending private clinics or hospitals [7]. Therefore, there is a need to evaluate if there is a delay in diagnosis among TB patients as this may hinder the efforts to control TB. A well designed health care seeking behavior study for TB patients and TB suspects would help to identify factors related to both patients and health system delay.

Our results has also shown that the diagnostic accuracy of the non-TB vignettes were also not optimum. This highlights the need for strategy that also incorporates other disease like PAL strategy (Practical Approach to Lung Health). The aim of this strategy is to increase the quality of the respiratory care in primary health care settings. Therefore, such strategy would improve the diagnosis and the management not only of TB patients but other conditions as well [18].

Private Public Mix (PPM) as a strategy has been recommended by the WHO. PPM has been shown to improve case detection rate and is a necessity for National TB Programs (NTP) to control TB [19]. In Oman, NTP should develop a contextualized strategy to engage with private GPs. Both private and public GPs should be actively engaged in TB control activities in a strategy of "Private Public Mix (PPM) for TB control in Oman". Such a strategy should explicitly highlight the rules for private sector in TB control, through implementing some of the practical tools recommended by the WHO, e.g. referral and notification as these are believed to increase case detection [19].

Although the study included only GPs working in Muscat governorate, the extrapolation of the results to Oman in general is valid as GPs in both the public and private sectors are not likely to be different from one region to another in Oman. One limitation of our study is that it did not assess the actual practice of the GPs, as vignettes are not appropriate to assess practice, they assess knowledge and competence. However, reported practice tends to be better than the actual practice. Therefore, the suspicion scores could be even lower than the reported.

## Conclusion

In Oman, general practitioners appear to have a low level of suspicion and knowledge of TB and private GPs have lower suspicion and knowledge compared to public. It is recommended to actively engage both private and public health sectors in the National TB Control Program training and the necessary educational activities.

## Competing interests

The authors declare that they have no competing interests.

## Authors' contributions

AAA–M was involved in study design, data collection, data analysis and Manuscript writing. OAA–R was involved in Study design, data analysis and manuscript writing. FA–A was involved in Study design, data collection and manuscript writing. ADC was involved in study design and manuscript writing. BE was involved in data analysis and manuscript writing. VKD was involved in Study design and Manuscript writing. All authors read and approved the final manuscript.

## Pre-publication history

The pre-publication history for this paper can be accessed here:



## Supplementary Material

Additional file 1 Clinical vignettes used in the survey. This file describes the clinical vignettes used to asses the suspicion of the GPs. Click here for file

Additional file 2 The 20 multiple choice questions questionnaire. This file has the 20 multiple choice questions used to assess the knowledge of the GPs. Click here for file
