# Supplementary material for: Tuberculosis suspicion and knowledge among private and public general practitioners: Questionnaire Based Study in Oman
Source: BMC Public Health. 2008 May 26;8:177. doi: 10.1186/1471-2458-8-177 (PMC2413224; doi:10.1186/1471-2458-8-177)
Supplement: Additional file 1 — Clinical vignettes used in the survey. This file describes the clinical vignettes used to asses the suspicion of the GPs. [file 1471-2458-8-177-S1.doc]

|  | Clinical Vignette | Details |
| --- | --- | --- |
| 1 | Sarcoidosis | Khalid, a 46-year-old man, complained of breathlessness on exercise for 6 months. He also had mild chest tightness and stiff joints but no skin or eye problems. There was no previous history of chest disease and he had never been abroad. On examination, he was afebrile and had bilateral basal inspiratory crackles. Rest of examination was unremarkable. His chest X-ray showed fine, diffuse shadows, predominantly in the mid zones, and bilateral hilar lymphadenopathy. |
| 2 | TB case 1 | Abdullah, 40 year old, works as a security guard in an industrial company in Muscat. He presents to the clinic with non-productive cough for 5 weeks associated with fever and loss of appetite for 2 weeks. On examination, his weight was 54 Kg and his temperature was 37.2 C. Remainder of examination was unremarkable except for mild pallor. |
| 3 | Fibrosis | Khadejah, a 59-year-old woman, complained of increasing exertional breathlessness for 2 years which rapidly worsened over the last 2 months. On examination she had finger clubbing and widespread crackles in her chest but no cyanosis or skin lesions. Her chest X-ray showed diffuse fine shadowing throughout both lung fields, especially in the lower zones |
| 4 | Pneumonia | Saeed, 32- year old man, presented with five-day history of acute shivering, cough, and pleuritic chest pain. He has no previous chest disease and never smoked. One examination, he looked ill with high grade fever (39.2°C), tachycardia (110/minute) and tachypnoea 28/minutes) and had course crackle in the left lower zone of his chest. |
| 5 | TB case 2 | Ali is 45-year old taxi driver was diagnosed as HIV positive 2 years ago. He presented with a 3-week history of cough and night sweats and one-week of a general feeling of malaise, but no haemoptysis, breathlessness or chest pain. On examination, he had features of anemia and was underweight, but his chest was clear. |
| 6 | TB case 3 | Fatma is 47-year old widow and living with her son presented with 3-month history of loss of appetite and general feeling of weakness. She has noticed that she is slightly febrile in the evenings for the last 2 weeks with occasional night sweats, and mild right chest pain with no other chest symptoms. On examination, she was febrile (37.8 C), with dullness and reduced breath sounds in the left lowers zone. Others systems were normal |
| 7 | Bronchiectasis | Zakiah is a 22-year old lady with history of productive cough since childhood, presented with one week history of fever, worsening productive cough and increasing breathlessness. Her younger brother has the same condition. On examination she was febrile, cyanosed with finger clubbing and had bilateral wheeze and crackles in her chest |
| 8 | TB case 4 | Moosa is a 31-year old from Muttrah. He has been unemployed for 2 years and been smoking cigarette and drinking alcohol since then. He presented with 3-week history of cough with heamoptysis in the last 2 days. He lost weight of 5 kgs over the last three months. On examination, he was malnourished and under-weight, but had no finger clubbing or cyanosis. His chest revealed course crackles in the right mid zone. |
| 9 | COPD | Salim is a 52-year old man with 7-year history of productive cough which worsens in winters, presented with 3 days history of increasing cough, sputum production and breathlessness. He stopped smoking 6 years ago. On examination, he was afebrile with no cyanosis or finger clubbing, but his chest was full of wheeze. |
| 10 | TB case 5 | Fahad is a 23-year-old man presented with a 4-week history of cough, breathlessness and general malaise. He had lost 4kg in weight, but had no history of night sweats or haemoptysis. On examination, he was mildly febrile (37.8°C) but had no evidence of anaemia or clubbing. Course crackles were audible over the apex of the left lung, with no other abnormalities. |

Box 1: Clinical Vignettes in the same order as presented in Questionnaire A.
