# Supplementary material for: Tuberculosis suspicion and knowledge among private and public general practitioners: Questionnaire Based Study in Oman
Source: BMC Public Health. 2008 May 26;8:177. doi: 10.1186/1471-2458-8-177 (PMC2413224; doi:10.1186/1471-2458-8-177)
Supplement: Additional file 2 — The 20 multiple choice questions questionnaire. This file has the 20 multiple choice questions used to assess the knowledge of the GPs. [file 1471-2458-8-177-S2.doc]

| Below are short case scenarios for Pulmonary TB patient. Each is followed by an MCQ with ***only one answer is right***. When you face an open question, It is not necessary to write in full sentences. Key words are enough. |
| --- |

**Salim is a 40 years old male from Muscat. He is married with 5 children. He presented with history of chronic cough. The general practitioner suspected pulmonary TB. On clinical examination, patient was normal. Then, the general practitioner asked for chest x-ray and AFB specimens to make diagnosis of pulmonary TB.**

According to Ministry of Health policy,

1. What is the minimum duration of cough required to suspect TB in this patient?

| 1. One week |  |
| --- | --- |
| 2. Two weeks |  |
| 3. Three weeks |  |
| 4. One month |  |
| 5. Others: Specify ……… |  |
| 6. Don’t Know |  |

2. How should AFB specimens be requested from the patient?

| 1. One specimen on the spot. |  |
| --- | --- |
| 2. Two specimens, one on the spot and one in the morning next day. |  |
| 3. Three specimens one on the spot and two early in the morning for tow consecutive days. |  |
| 4 Three specimens all should be early in the morning for 3 consecutive days. |  |
| 5. Others: Specify…………………… |  |
| 6. Don’t Know |  |

3. What is the definition of Smear Positive?

| 1. | Patient with at least two sputum specimens positive |  |
| --- | --- | --- |
| 2. | Patient with one sputum specimen positive and x-ray is suggestive |  |
| 3. | Patient with at least one sputum specimen positive and culture positive |  |
| 4. | Any of the above |  |
| 5. | Others specify ……… |  |
| 6. | Don’t Know |  |

4. What other underlying infection should this patient is routinely screened for?

| 1. Pneumonia |  |
| --- | --- |
| 2. Atypical mycobacterium |  |
| 3. HIV |  |
| 4. Others: specify…………. |  |
| 5. Don’t Know |  |

**The case was notified and then referred to a hospital for Anti-TB treatment. The patient was told that he will be admitted in the hospital and he will be put on standard DOTS regimen.**

According to Ministry of Health Policy,

5. When should Pulmonary TB case notified? (Select from below)

| 1. Within 24 hours |  |
| --- | --- |
| 2. Within one week |  |
| 3. Within two weeks |  |
| 4. Within one month |  |
| 5. Others. Specify…………. |  |
| 6. Don’t Know |  |

6. How long should this patient be admitted?

| 1. One week |  |
| --- | --- |
| 2. Two week |  |
| 3. One month |  |
| 4. Two months |  |
| 5. Others: specify………. |  |
| 6. Don’t Know |  |

7. What is the main purpose of patient admission?

| 1. Insure that patient take medications |  |
| --- | --- |
| 2. Isolate the patient |  |
| 3. Monitor sputum smear |  |
| 4. Monitor the side effects of the drugs |  |
| 5. Other: specify…………………….. |  |
| 6. Don’t Know |  |

**The patient was admitted at the hospital and started the initial phase of the treatment.**

8. How long is the initial phase?

| 1. Two weeks |  |
| --- | --- |
| 2. One month |  |
| 3. Two months |  |
| 4. Others: specify…………. |  |
| 5. Don’t Know |  |

9. What are the **four** standard drugs used in the initial phase in Oman? (Please select from the following list)

| 1. Caperomycin |  | 5. Pyrazinamide |  |
| --- | --- | --- | --- |
| 2. Ethambutol |  | 6. Prothinamide |  |
| 3. D-cyclosrine |  | 7. Rifampicin |  |
| 4. Isoniazaid |  | 8. Streptomycin |  |

10. Which blood test is essential before starting the drugs?

| 1. CBC |  |
| --- | --- |
| 2. Thyroid function test |  |
| 3. Renal Function Test |  |
| 4. Liver Function Test |  |
| 5. Others: specify………. |  |
| 6. Don’t Know |  |

**After completion of initial phase, he was referred to the local health centre for the continuation phase.**

11. How long is the continuation phase?

| 1. Two weeks |  |
| --- | --- |
| 2. One month |  |
| 3. Two months |  |
| 4. Others: specify…………. |  |
| 5. Don’t Know |  |

12. What are the **two** standard drugs used in the continuation phase? (Please select from the following list)

| 1. Caperomycin |  | 5. Pyrazinamide |  |
| --- | --- | --- | --- |
| 2. Ethambutol |  | 6. Prothinamide |  |
| 3. D-cyclosrine |  | 7. Rifampicin |  |
| 4. Isoniazaid |  | 8. Streptomycin |  |

13. To assess the treatment outcome, what would be the single most important investigation preformed during continuation phase?

| 1. ESR |  |
| --- | --- |
| 1. Chest x-ray |  |
| 2. Mantoux |  |
| 3. Sputum culture |  |
| 5. Others: specify………… |  |
| 6. Don’t Know |  |

14. How often will you do the above investigation?

| 1. Every week |  |
| --- | --- |
| 2. Every two weeks |  |
| 3. Every Three weeks |  |
| 4. Every Month |  |
| 5. Others: Specify……… |  |
| 6. Don’t Know |  |

**As part of contact tracing, the wife of the above patient was reported to be asymptomatic and had a normal chest x-ray. She was reassured and asked to report back if she became unwell in anyway.**

According to Ministry of Health Policy,

15. Of patient contacts, who should be investigated to rule out TB?

| 1. | All household contacts |  |
| --- | --- | --- |
| 2. | Only living with patient in the same room |  |
| 3. | Only symptomatic contacts |  |
| 4. | Only Children contacts with no BCG scar |  |
| 5. | Others: Specify………… |  |
| 6. | Don’t Know |  |

16. Who are the contacts eligible for TB prophylaxis among adults?

| 1. | All adult contacts |  |
| --- | --- | --- |
| 2. | Adults with cough |  |
| 3. | Adults more than 50 years old |  |
| 4. | Not indicated |  |
| 5. | Others specify……………….. |  |
| 6 | Don’t Know |  |

17. Who are the contacts eligible for TB prophylaxis among children?

| 1. | All children |  |
| --- | --- | --- |
| 2. | Children less than 3 years |  |
| 3. | Children more than 6 years |  |
| 4. | Not indicated |  |
| 5. | Others. Specify……………… |  |
| 6 | Don’t Know |  |

18. What is the standard drug used for prophylaxis?

| 1. Caperomycin |  | 5. Pyrazinamide |  |
| --- | --- | --- | --- |
| 2. Ethambutol |  | 6. Prothinamide |  |
| 3. D-cyclosrine |  | 7. Rifampicin |  |
| 4. Isoniazaid |  | 8. Streptomycin |  |

19. After how long do patients with smear positive treated with standard ani-TB drugs become non-infectious, in general?

| 1. One week |  |
| --- | --- |
| 2. Two weeks |  |
| 3. One month |  |
| 4. Two months |  |
| 5. Others: specify………. |  |
| 6. Don’t Know |  |

20. When should the patient be labeled as cured?

| 1. | Patient completed the treatment. |  |
| --- | --- | --- |
| 2. | Patient completed the treatment and had the last smear as sputum negative prior stopping treatment. |  |
| 3. | Patient completed the treatment and had at least two negative smear results during the treatment. |  |
| 4. | Patient completed the treatment and had at least two negative smear results during the continuation phase prior stopping the treatment |  |
| 5. | Others specify……………………………………. |  |
| 6. | Don’t Know |  |
